# Purinergic Signalling in the Cochlea

**DOI:** 10.3390/ijms232314874

**Published:** 2022-11-28

**Authors:** Srdjan M. Vlajkovic, Peter R. Thorne

**Affiliations:** 1 Department of Physiology and The Eisdell Moore Centre, Faculty of Medical and Health Sciences, The University of Auckland, Private Bag 92019, Auckland 1142, New Zealand; 2 Section of Audiology, School of Population Health, University of Auckland, Private Bag 92019, Auckland 1142, New Zealand

**Keywords:** ATP, P2X receptors, P2Y receptors, ectonucleotidases, adenosine, adenosine receptors, cochlea, hearing loss

## Abstract

The mammalian cochlea is the sensory organ of hearing with a delicate, highly organised structure that supports unique operating mechanisms. ATP release from the secretory tissues of the cochlear lateral wall (stria vascularis) triggers numerous physiological responses by activating P2 receptors in sensory, supporting and neural tissues. Two families of P2 receptors, ATP-gated ion channels (P2X receptors) and G protein-coupled P2Y receptors, activate intracellular signalling pathways that regulate cochlear development, homeostasis, sensory transduction, auditory neurotransmission and response to stress. Of particular interest is a purinergic hearing adaptation, which reflects the critical role of the P2X_2_ receptor in adaptive cochlear response to elevated sound levels. Other P2 receptors are involved in the maturation of neural processes and frequency selectivity refinement in the developing cochlea. Extracellular ATP signalling is regulated by a family of surface-located enzymes collectively known as “ectonucleotidases” that hydrolyse ATP to adenosine. Adenosine is a constitutive cell metabolite with an established role in tissue protection and regeneration. The differential activation of A_1_ and A_2A_ adenosine receptors defines the cochlear response to injury caused by oxidative stress, inflammation, and activation of apoptotic pathways. A_1_ receptor agonism, A_2A_ receptor antagonism, and increasing adenosine levels in cochlear fluids all represent promising therapeutic tools for cochlear rescue from injury and prevention of hearing loss.

## 1. Introduction

**Figure 1:**
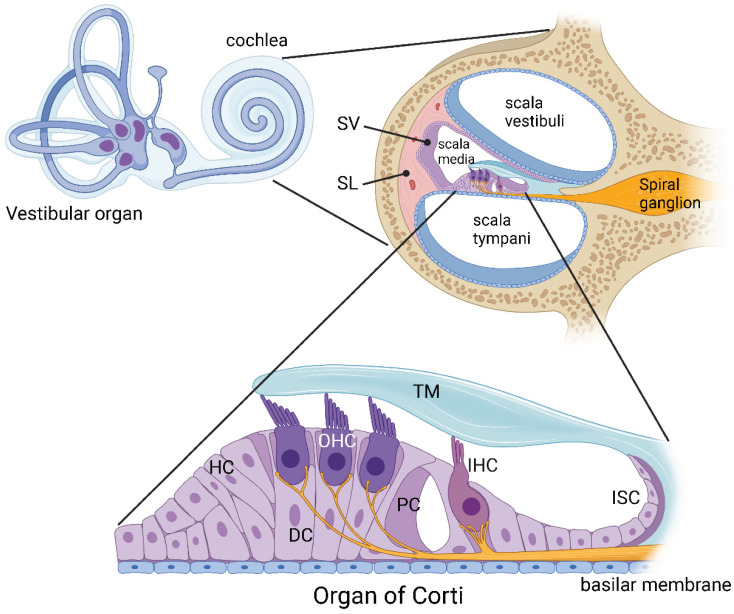
**Inner ear anatomy.** The mammalian inner ear contains two sensory organs, the organ of hearing (cochlea) and the organ of balance (vestibular organ). The cochlea comprises three fluid-filled compartments. Scala vestibuli and scala tympani contain Na^+^-rich perilymph, whereas scala media contains K^+^-rich endolymph. The lateral wall of the cochlea comprises the spiral ligament (SL) made up of fibrocytes, providing structural support to the secretory tissues of the stria vascularis (SV). The organ of Corti is the central structure for sensory transduction in the cochlea. It is covered by the tectorial membrane (TM) and contains two types of sensory cells, inner hair cells (IHC) and outer hair cells (OHC). These sensory cells are surrounded by supporting cells, such as Deiters’ cells (DC), Hensen’s cells (HC) and pillar cells (PC). The inner sulcus cells (ISC) are epithelial cells that form the medial border of the organ of Corti. Created with BioRender.com.

The cochlea of the inner ear provides exquisite sensitivity and frequency selectivity to sound detection in the mammalian auditory system [1]. It contains three fluid-filled compartments: scala media filled with endolymph (high K^+^ and low Na^+^ concentration), scala vestibuli and scala tympani filled with perilymph (high Na^+^ and low K^+^). In the mammalian cochlea, the sensory epithelium of the organ of Corti contains sensory hair cells surrounded by various supporting cells, including Deiters’, Hensen’s and pillar cells [2] (Figure 1). Inner hair cells (IHC) are responsible for sound transduction (conversion of acoustic stimuli to electrical signals to the brain), whilst outer hair cells (OHC) are known as “cochlear amplifiers”, providing enhanced frequency selectivity and sensitivity at low sound levels [3]. Primary auditory neurons located in the spiral ganglion provide afferent innervation mostly to the IHC, and the efferent innervation from the brainstem structures (superior olivary complex) modifies the activity of the OHC [2]. Cochlear supporting cells are essential constituents of an epithelial network that regulates the unique ionic composition of cochlear extracellular fluids [4]. The spiral ligament and stria vascularis form the lateral wall of the cochlea and contribute to the maintenance of ion homeostasis, generation of the endocochlear potential (a positive electrical potential confined to scala media), and regulation of cochlear metabolism [2]. 

Amongst the multiple signaling pathways that regulate cochlear function, significant attention has been focused on purinergic regulation, particularly its protective adaptation to acoustic stimuli [5,6]. Adenosine 5′-triphosphate (ATP) release from marginal cells in the stria vascularis [7] exerts a homeostatic regulatory mechanism promoting K^+^ efflux from scala media and reduction of the endocochlear potential, thus reducing cochlear sensitivity to loud sound [8,9]. Purinergic signalling in the cochlea can affect several essential functions, from ion homeostasis to active mechanical amplification by the outer hair cells [10].

The release of ATP and activation of purinergic receptors in cochlear sensory, supporting, secretory and neural tissues play a critical role in cochlear physiology and pathophysiology [11,12]. The ionotropic P2X and the metabotropic P2Y receptors are widely distributed in cochlear tissues [6,13]. To date, seven subtypes of the P2X family (P2X_1–7_) and eight subtypes of the P2Y family (P2Y_1,2,4,6,11–14_) have been cloned and functionally characterised [12]. P2X receptors provide direct routes for Ca^2+^ entry into the cell, whereas P2Y receptors either activate phospholipase C (PLC) and release intracellular Ca^2+^ or alter cAMP levels [12,14]. Intracellular Ca^2+^ seems to be the principal second messenger in ATP-mediated signaling [15].

P2 receptors have been implicated in the sensory cell sensitivity adjustment, regulation of cochlear electrochemical homeostasis, blood flow, and cochlear development [6]. P2 receptor signalling is regulated by ectonucleotidases, which hydrolyse extracellular ATP to adenosine [16,17]. Adenosine activates protective mechanisms in the cochlea by acting on adenosine (P1) receptors expressed on cochlear sensory and non-sensory cells [13,18]. 

A substantial body of data suggests that extracellular ATP is a critical player in processes that guide the activation of the mammalian auditory system in response to sound during ear development. Spontaneous activity in afferent auditory pathways appears before the onset of acoustically evoked signal processing [19,20]. During this critical developmental period, IHC fire bursts of action potentials that stimulate synaptic maturation and refinement of auditory circuits to establish a precise tonotopic organization in the developing cochlea and receptive fields in central auditory nuclei [19,20]. Endogenous ATP release in the organ of Corti triggers this activity by activating diverse P2 receptors in supporting cells and the IHC. ATP initiates Ca^2+^ waves spreading through the developing cochlea, causing a K^+^ efflux from these cells through Ca^2+^-activated K^+^ channels and the leak K^+^ channels [19]. 

P2 receptor signalling contributes to neuronal firing by increasing the excitability of auditory nerve fibers, spiral ganglion and auditory brainstem neurons [19,20]. ATP release during cochlear development is central to this process. The neonatal cochlea has a transient epithelium (the Kölliker’s organ) which spontaneously secretes ATP in rhythmical bursts that activate P2 receptors on the IHC [21]. This, in turn, synchronises the output of adjacent IHC, which may refine tonotopic maps in the brain auditory nuclei.

Several lines of evidence also support the notion that ATP-dependent mechanisms safeguard against the harmful effects of acoustic overstimulation in the adult cochlea [8]. Purinergic-mediated adaptation of the cochlear sensory structures to loud sound decreases the activity of the sensory cells and protects them and their afferent synapses from sustained noise exposure and acoustic injury [22]. 

This review highlights the role of purinergic P2 (ATP) and P1 (adenosine) receptors in regulating cochlear function in health and disease, focusing on the recent developments in the field. 

## 2. Methodology

A literature search of relevant publications from 2000 to 2022 was performed using PubMed and Google Scholar. We have also used reference lists from the relevant papers. Following Boolean search logic, we used the following keywords: (cochlea) and (ATP OR P2 receptors OR adenosine receptors OR purinergic signalling OR ATP release OR ectonucleotidases OR adenosine transport); (ATP OR P2 receptors) and (cochlear development); (vestibular system) and (P2 receptors OR purinergic signalling). The search results were then examined according to their relevance to this review. Only English language publications were included in this review.

## 3. ATP Release in the Cochlea

Various mechanisms are involved in ATP release from cells, including exocytosis, transporter proteins, and ion channel-mediated release [23]. Tissue injury can also lead to lytic release from damaged cells, rapidly raising the extracellular ATP concentrations [23]. 

The principal conduits for ATP release in the cochlea during development and adulthood are integral membrane proteins from the gap junction family known as pannexin and connexin hemichannels [24]. The opening of these channels allows the efflux of ATP down its concentration gradient and release into the endolymph and perilymph [24,25,26]. Connexin and pannexin hemichannels are essential for the cellular release of ATP and regulate purinergic receptor activation [25]. In turn, P2 receptor activation by ATP can amplify purinergic signaling through a positive feedback loop via inositol 1,4,5-trisphosphate (IP_3_), giving rise to the concept of ATP-induced ATP release [27]. IP_3_, activated by P2Y receptors, mobilises intracellular Ca^2+^ which opens pannexin hemichannels, enabling the tide of calcium waves across epithelial cells [28]. 

Several P2X receptor subtypes (P2X_2_, P2X_4_, P2X_7_) can also activate the pannexin 1 (Panx1) channel to carry through large molecules involved in initiating inflammatory responses and apoptotic cell death [28,29].

**Figure 2:**
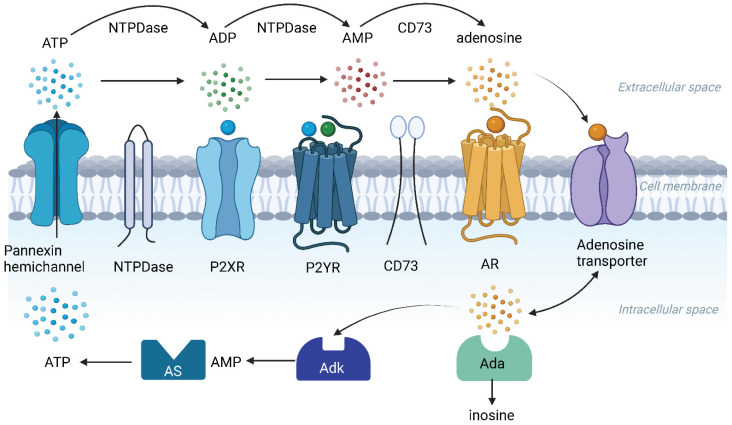
**The elements of the purinergic signalling system in the cochlea.** Pannexin hemichannels (e.g., Panx1) are the principal conduits of ATP release in the cochlea. Extracellular ATP activates ATP-gated ion channels (P2X receptors) and G protein-coupled receptors (P2Y receptors). ATP is hydrolysed to adenosine 5′-diphosphate (ADP) and adenosine 5′-monophosphate (AMP) by surface-located ecto-nucleoside triphosphate diphosphohydrolases (NTPDases), whereas AMP is dephosphorylated to adenosine by ecto-5′-nucleotidase (CD73). Adenosine activates four types of adenosine receptors (AR: A_1_, A_2A_, A_2B_ and A_3_). Bi-directional adenosine transporters mediate cellular uptake of adenosine. In the intracellular space, adenosine is hydrolysed to inosine by adenosine deaminase (Ada) or phosphorylated to AMP by adenosine kinase (Adk) and ATP by ATP synthase (AS). Created with BioRender.com.

Pannexins have different electrophysiological and pharmacological properties than connexins [30]. Three isoforms of the pannexin hemichannel (Panx1, 2 and 3) have been identified in the cochlea, mostly in the supporting cells, the spiral limbus, and the lateral wall [31]. Panx1 is a dominant pannexin isoform in the cochlea [24]. *Panx1* deletion abolishes cochlear ATP release and ATP-mediated K^+^ cycling essential for maintaining the endocochlear potential (EP) in the mammalian cochlea [32,33]. EP is a driving force for hair cell transduction and is essential for normal hearing. *Panx1* deficiency causes moderate-to-severe progressive hearing loss and the progressive loss of sensory hair cells by activating the caspase-3 apoptotic pathway [33]. Interestingly, the deletion of predominant connexin isoforms in the cochlea, connexin 26 (*Cx26*) and connexin 30 (*Cx30)*, does not reduce ATP release under physiological conditions, suggesting that Panx1 channels dominate ATP release in the cochlea [24] (Figure 2).

## 4. P2 Receptors in the Cochlea

### 4.1. P2X Receptors in the Cochlea

All P2X receptors are transiently expressed in the developing mammalian cochlea, but their immunoexpression is limited in the adult cochlea [6,13].

P2X_1_ receptor (P2X_1_R) is transiently expressed in the otic capsule, spiral limbus, epithelial cells of the Reissner’s membrane and spiral ganglion neurons (SGN) during early postnatal development in rats but is absent after hearing onset [34].

P2X_2_ receptor (P2X_2_R) is the predominant P2X subtype expressed in the epithelial cells lining the cochlear partition of the rat and mouse cochlea, including the inner and outer sensory hair cells, supporting Deiters’ cells in the organ of Corti and Reissner’s membrane that separates endolymph in scala media from perilymph in scala vestibuli [35,36]. 

P2X_3_ receptor (P2X_3_R) is also transiently expressed in the developing cochlear tissues. Its expression is detected in perinatal and juvenile C57BL/6 mice from embryonic day 18 (E18) to postnatal day 6 (P6) in the SGN, sensory hair cells, and peripheral neurites projecting towards the sensory hair cells [37]. The P2X_3_R immunoexpression in the peripheral neurites and the hair cells diminishes by P6 and is absent after the onset of hearing (P11 to P17) [37]. The transient expression of these receptors, particularly P2X_3_R, follows a precise spatio-temporal profile suggesting the role of this receptor in synaptic pruning [37]. The synaptic pruning likely involves transiently expressed heterodimeric P2X_2/3_ receptors inhibiting neurotrophic support for SGN during synaptic reorganisation [38].

In the adult guinea pig cochlea, P2X_4_R is expressed in the outer hair cells [39] and spiral ligament capillaries [40], the latter suggesting that extracellular ATP regulates blood flow in the cochlear lateral wall by activating P2X_4_R in endothelial cells.

There is little evidence for the immunoexpression of P2X_5_ and P2X_6_ receptors in the mammalian cochlea [41].

All P2X receptors are expressed in the developing rat spiral ganglion, but only P2X_2_ and P2X_7_ are sustained into adulthood [6]. P2X_2_ was immunolocalised to the postsynaptic membranes of Type I and Type II SGN [35,42]. Strong P2X_7_ expression was also observed in the olivocochlear efferent fibres innervating the sensory hair cells from E18 through to adulthood, suggesting a role for these receptors in auditory neurotransmission [43]. Recent evidence, however, indicates that P2X_7_R is immunolocalised to peripheral glial cells rather than afferent neurons in the auditory nerve of small rodents [44]. Physiological responses in the peripheral glia are characterised by classical features of P2X_7_R activation, including the formation of ion- and macromolecule-permeable pores. These properties suggest that P2X_7_R could contribute to glial-mediated inflammatory processes under pathologic conditions, potentially contributing to auditory neuropathy and hearing loss [44].

With sustained elevated sound levels, ATP is released into the endolymph and ATP-gated ion channels (P2X receptors) on the epithelial cells lining the endolymphatic compartment shunt K^+^ outside scala media, reducing the driving force for sensory transduction and contributing to protective hearing adaptation [8]. The P2X_2_ receptor subunit on Reissner’s membrane and other epithelial tissues lining the cochlear endolymphatic compartment is essential for this shunt conductance evoked by noise exposure [45].

**Figure 3:**
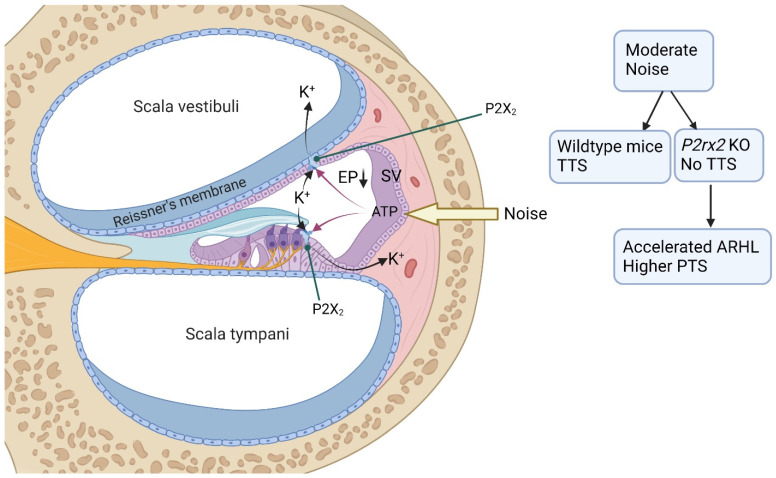
**Purinergic hearing adaptation.** Exposure to moderate noise levels induces ATP release from the stria vascularis (SV). ATP activates P2X_2_ receptors in the tissues lining the endolympatic compartment (e.g., Reissner’s membrane, sensory and supporting cells). The activation of P2X_2_ receptors mediates K^+^ shunt conductance from scala media, thus reducing the endocochlear potential (EP), the driving force for sensory transduction. Reduced EP results in decreased sensitivity of the cochlea to sound stimulation, protecting the ear from permanent damage and hearing loss. The role of P2X_2_ receptors in hearing adaptation was demonstrated using *P2rx2* knockout (KO) mice. Exposure to moderate noise levels caused temporary threshold shift (TTS) in wildtype mice but not in *P2rx2* KO mice. Without purinergic hearing adaptation, *P2rx2* KO mice showed accelerated age-related hearing loss (ARHL) and elevated permanent threshold shift (PTS) in response to higher noise levels. Created with BioRender.com.

A significant component of temporary hearing loss that develops with sustained exposure to moderate noise has been attributed to the release of ATP in the cochlea, activating the P2X_2_ receptor in scala media [8]. This purinergic hearing adaptation enables the cochlea to detect sounds in background noise and may also protect the cochlea from permanent damage and hearing loss (Figure 3). In the study that established this hearing adaptation mechanism [8], the role of the P2X_2_ receptor was determined in *P2rx2* knockout and age-matched wildtype mice using auditory brainstem responses (ABR) measured during sustained noise exposure. The knockout mice failed to exhibit the temporary threshold shift (TTS) observed in wildtype mice after exposure to sustained moderate noise levels (85 dB SPL). This finding was a paradigm shift in understanding the mechanism of TTS, as the study demonstrated that the P2X_2_R almost exclusively mediated TTS under moderate noise conditions. In the absence of TTS, the *P2rx2* KO mice exhibited normal hearing sensitivity at a young age but developed accelerated age-related hearing loss compared to wildtype mice. In addition, *P2rx2* KO mice demonstrated increased vulnerability to sustained loud sound at higher noise levels (95 dB SPL), developing significantly higher permanent threshold shifts (PTS) than wildtype mice [8,22,46]. 

The study by Housley et al. [8] complements a report by Yan et al. [47], which demonstrated that the absence of cochlear P2X_2_R signaling in two Chinese families due to a dominant-negative mutation (conversion of 178G>T (p.V60L)) at chromosome 12, removed intrinsic purinergic otoprotection and induced the autosomal-dominant progressive hearing loss designated as DFNA41. Members of the family with DFNA41 with a history of noise exposure demonstrated enhanced high-frequency hearing loss, previously modelled in *P2rx2*-null mice [8]. Another knock-in mouse model based on human p.V60L mutation exhibited hearing loss at 21 days of age and progressed to deafness by six months [48]. Abnormal morphology of the inner hair cells and ribbon synapses was observed in those mice [48]. Other studies demonstrated that mutations in human P2X_2_R could cause hearing loss without completely disrupting channel function [49], highlighting the important role of these receptors in hearing protection.

Hearing protection is also regulated by ATP-evoked Ca^2+^ signaling in the supporting cells of the organ of Corti [50]. Extracellular ATP controls the intercellular Ca^2+^ waves, which travel through supporting cells regulating the repair mechanisms following acoustic trauma [15]. Lahne and Gale [51] showed that two distinct Ca^2+^ waves are triggered during cochlear damage in organotypic tissue cultures, both elicited by extracellular ATP. A slower Ca^2+^ wave in Deiters’ cells was mediated by P2Y receptors and Ca^2+^ release from IP_3_-sensitive stores. The faster Ca^2+^ wave propagated through sensory hair cells and was likely mediated by the P2X_4_ receptor [51]. Periodic Ca^2+^ waves have been linked to gene regulation and likely play a crucial role in developing the organ of Corti and the acquisition of hearing [52]. 

Liu et al. [53] have shown that type II unmyelinated cochlear afferents that innervate OHC are activated when OHC are damaged. This response depends on both P2X and P2Y receptors and is activated by ATP released from nearby supporting cells in response to hair cell damage. Type II afferents may thus represent cochlear nociceptors, and their activation may reflect evasion of further injury to the inner ear after irreversible damage to OHC [53].

### 4.2. P2Y Receptors in the Cochlea

The immunoexpression of the P2Y receptors (P2Y_1_, P2Y_2_, P2Y_4_, P2Y_6_, and P2Y_12_) was demonstrated in the developing (E16-P28) and the adult rat cochlea (P49-P56) by Huang et al. [54]. In the sensory epithelium, the earliest expression of P2Y receptors (P2Y_2_ and P2Y_4_) was observed in the greater epithelial ridge at embryonic day 18 (E18), and this expression pattern was retained at birth (P0). At P0, the P2Y_6_ receptor was immunolocalised to the immature IHC and OHC [54], whereas at the early postnatal age (P6–P12), the P2Y_6_ receptor localisation resembled the immunoexpression in adults. In adult rats, P2Y_6_ becomes the predominant subtype in the IHC, and both P2Y_1_ and P2Y_4_ receptors are immunolocalised to the OHC [54]. The predominant P2Y receptor in the supporting cells is the P2Y_2_ receptor immunolocalised to Deiters’ cells, Hensen’s cells, pillar cells and Claudius’ cells, supporting cells that have a role in intercellular communication [51,54].

P2Y receptors (P2Y_2_, P2Y_4_, P2Y_6_, and P2Y_12_) were detected in the SGN at birth except for P2Y_1_, which was expressed later in the postnatal age, and this expression pattern was retained until adulthood [54]. In the lateral wall tissues, P2Y receptor expression was first observed at early postnatal age (P0-P6), with P2Y_1_ and P2Y_2_ as the predominant subtypes. Following the onset of hearing, the P2Y expression in the lateral wall shifted to P2Y_2_ and P2Y_4_ [54]. This distribution of purinergic P2Y receptors suggests their multiple roles in cochlear development, maintaining cochlear homeostasis, and regulating sound transduction and neurotransmission [6]. 

Interestingly, pharmacological inhibition of the P2Y_1_ receptor dramatically reduces spontaneous activity in the developing cochlea [55]. Spontaneous bursts of electrical activity in the developing auditory system arise within the cochlea before hearing onset to promote the maturation of auditory neurons. ATP release from supporting cells and activation of P2Y_1_ receptors invokes coordinated excitation of neurons that will process similar sound frequencies [55]. The role of P2Y receptors in cochlear development is discussed further in the next section.

## 5. ATP and Cochlear Development

From birth to hearing onset, the auditory system relies on intrinsic mechanisms that elicit the coordinated firing of neurons processing similar sound frequencies in the adult cochlea [20,21]. ATP is released from the supporting cells in the greater epithelial ridge (GER) of the neonatal rat cochlea, a transient non-sensory cell population that disappears during postnatal cochlear maturation. Lysosomes are the organelles involved in ATP storage and release from GER cells [56]. ATP is also stored in the stria vascularis, and ATP-containing vesicles in marginal cells have also been identified as lysosomes [57]. ATP release from marginal cells and GER involves Ca^2+^-dependent lysosomal exocytosis [56,57]. Lysosomal exocytosis of ATP is coupled to the P2Y_2_ receptor in marginal cells via the P2Y_2_R-phospholipase C-IP_3_ pathway [58]. Connexin hemichannels mediate the release of ATP responsible for Ca^2+^ wave propagation in the developing mouse cochlea [58].

Before the onset of hearing, immature IHC and primary auditory neurons in the spiral ganglion experience sound-independent activity, which is believed to be important in retaining and refining neural connections in the absence of sound [21]. This activity originates in a group of transient epithelial supporting cells forming Kölliker’s organ (as part of GER), which is only present during cochlear development. ATP released through connexin hemichannels may activate P2 receptors in both Kolliker’s organ and the adjacent IHC, leading to the generation of electrical activity in the auditory system [59,60]. It was proposed that inner border cells have a major role in generating spontaneous morphological activity within Kölliker’s organ [60,61]. 

More recently, it was proposed that Ca^2+^ waves in the supporting GER cells cause increased and synchronized Ca^2+^ activity in the OHC via ATP-induced activation of P2X_3_ receptors [62]. This synchronization is required for the refinement of their immature afferent innervation. Ceriani et al. proposed that the correct maturation of the afferent connectivity of OHCs requires sound-independent Ca^2+^ signalling from sensory and non-sensory cells, which was P2X_3_ receptor-dependent [62].

At all developmental stages, pharmacological inhibition of the P2Y_1_ receptor dramatically reduces spontaneous activity in sensory and non-sensory cells [55]. The frequency of the spontaneous activity increases progressively during the postnatal prehearing period but remains dependent on the P2Y_1_R located on the cochlear supporting cells. When P2Y_1_R is activated, it triggers the release of Ca^2+^ in supporting cells and the activation of Ca^2+^-dependent potassium channels. The efflux of K^+^ to the extracellular space activates the sensory hair cells, but it also causes supporting cells to shrink due to water egress [63]. Conversely, when P2Y_1_R is inhibited, this causes the supporting cells to swell, entrapping potassium ions near the sensory cells. Bursts of electrical activity are thus controlled by the rhythmic swelling and shrinking of supporting cells mediated by the P2Y_1_R [63].

Other P2 receptors may also be involved in cochlear maturation. Outer sulcus cells (OSC) adjacent to the lateral wall of the cochlea may have a role in maintaining an adequate K^+^ concentration in the cochlear endolymph in response to variable intensities of auditory stimulation [64]. Temporal changes in P2Y_4_R expression during OSC development likely contribute to the endolymphatic ion composition required to generate the endocochlear potential through the activation of K^+^ channels [64]. 

In addition, P2X_3_ receptors may be required for the differentiation of Type I SGN and their branch refinement [65]. Synaptic refinement and strengthening are activity-dependent processes aiding the orderly arrangement of cochleotopic maps in the central auditory system. The maturation of auditory brainstem circuits is guided by the electrical activity of the IHC in the developing cochlea, and modulated by paracrine ATP signalling [66]. Using slice recordings before hearing onset and in vivo recordings after hearing onset, Jovanovic et al. showed that cell-specific purinergic modulation follows a precise tonotopic pattern in the ventral cochlear nucleus in gerbils, which was mediated by the heterologous P2X_2/3_ receptor [66]. 

## 6. Ectonucleotidases in the Cochlea

Ectonucleotidases are a large family of surface-located enzymes that hydrolyze extracellular nucleotides (ATP, UTP) to their respective nucleosides and thus regulate complex extracellular P2 receptor signalling pathways in mammalian tissues [16,67]. The best-characterised ectonucleotidase family in the mammalian cochlea is the ecto-nucleoside triphosphate diphosphohydrolase (NTPDase) family [18]. All enzymes from this family (NTPDase1-8) are expressed in the adult rat cochlea (Figure 2). The spatial and temporal expression of NTPDases in various cell types in the vasculature, sensory and neural tissues in the cochlea impacts multiple physiological and pathophysiological processes, including cochlear response to noise [68]. 

Vlajkovic et al. provided a detailed description of NTPDase1 and NTPDase2 distribution in mouse and rat cochlear tissues using immunocytochemistry [17,69]. These two cell surface-located enzymes have different hydrolytic profiles: NTPDase1 hydrolyses nucleoside 5′- triphosphates (NTPs) and nucleoside 5′-diphosphates (NDPs) to a similar extent, whilst NTPDase2 has a high preference for NTPs [67]. NTPDase1 immunoexpression was most prominent in the cochlear vasculature and cell bodies of the spiral ganglion neurons, whereas considerable NTPDase2 immunoreactivity was detected in the stria vascularis [17,69]. Both NTPDases were localised in the cuticular plates of the sensory hair cells, and auditory nerve fibres projecting from the synaptic area underneath the inner and outer hair cells. Their localisation corresponds to the reported distribution of the P2X_2_ receptor in sensory, supporting and neural cells and P2Y receptor distribution in the cochlear vasculature and secretory tissues of the lateral wall. The putative role of NTPDase1 and 2 in the cochlea is to regulate extracellular ATP signalling involved in cochlear blood flow, electrochemical regulation of sound transduction and neurotransmission in the cochlea [17,69].

NTPDase3 (NTPase activity > NDPase) immunoreactivity was observed in the primary afferent neurons of the spiral ganglion and their neurites extending to the synapses beneath the inner and outer hair cells, suggesting a role for NTPDase3 in regulating ATP signaling associated with auditory neurotransmission [70]. Semi-quantitative immunohistochemistry revealed increased NTPDase3 immunolabeling in the synaptic regions of the inner and outer hair cells at elevated sound levels. NTPDase3 upregulation in the noise-exposed cochlea can prevent the activation of the cytotoxic P2X_7_ receptor, suggesting the potential neuroprotective nature of this ectonucleotidase [70].

O’Keeffe et al. reported the dynamic changes in the expression of NTPDase5 and 6 in the developing and adult rat cochlea [71,72]. These two intracellular members of the NTPDase family can be released in a soluble form and show a preference for nucleoside 5’-diphosphates, such as uridine 5′-diphosphate (UDP) and guanosine 5′-diphosphate (GDP). NTPDase6 immunolocalisation in the developing cochlea underpins its putative role in hair cell bundle development, while NTPDase5 may have an extracellular role in the development of sensory and neural tissues [72]. In the adult rat cochlea, upregulation of NTPDase5 after exposure to loud sound indicates a possible role for NTPDase5 in cochlear response to stress [71]. In addition, NTPDase6 immunolocalisation in the vestibular end organ could be linked to the maintenance of vestibular hair bundles [73].

P2 receptors in the cochlea initiate various signaling pathways that could be involved in the noise-induced cochlear injury. Stimuli such as noise or hypoxia could induce the excessive release of ATP into the cochlear fluid spaces [7], which may exert a cytotoxic effect mainly acting on the P2X_7_ receptor. Membrane-bound NTPDases appear essential for regulating extracellular nucleotide concentrations and P2 receptor signaling in the cochlea in physiological and pathophysiological conditions. In the rat cochlea exposed to traumatic noise (110 dB SPL), we observed increased expression of NTPDase1 and NTPDase2 mRNA transcripts, while mild noise (90 dB SPL) altered only NTPDase1 mRNA expression levels [68]. Functional studies revealed increased ATPase activities in the cochlea after exposure to traumatic noise, consistent with the up-regulation of NTPDases. The changes in NTPDase expression may reflect the adaptive response of cochlear tissues to limit ATP signaling during noise exposure and thus protect the cochlea [68].

## 7. Adenosine Receptor Signalling in the Cochlea

Adenosine is a naturally occurring purine nucleoside that mediates its physiological actions by interacting with four cell surface-located adenosine receptors (A_1_, A_2A_, A_2B_, A_3_) distributed throughout the body [74]. Adenosine can be released from cells via specific bi-directional adenosine transporters and is also the end-product of ATP hydrolysis (Figure 2). Adenosine receptors are G protein-coupled receptors activating diverse cellular signaling pathways that define their tissue-specific roles [75]. The specific tissue distribution of adenosine receptors in the mammalian cochlea implicates the role of adenosine signalling in cochlear blood flow, sensory transduction and auditory neurotransmission [18]. The ability of adenosine A_1_ receptors to reduce oxidative stress and inflammation in the cochlea and thus prevent cochlear injury caused by acoustic trauma or ototoxic drugs has opened a new chapter in the preventative treatment of sensorineural hearing loss [18,76]. The balance between A_1_ and A_2A_ receptors appears to be a critical factor for cochlear response to oxidative stress, which has been established as an underlying mechanism of several inner ear pathologies (e.g., noise-induced, age-related and drug-related hearing loss) [77]. Preclinical studies have demonstrated the extraordinary potential of adenosine receptor ligands (agonists and antagonists) in regulating the cochlear response to stress and injury and opened new avenues for the pharmacological management of hearing loss [18].

The distribution of A_1_, A_2A_, A_3_ receptors was identified by immunohistochemistry [78,79]. Adenosine receptors were differentially expressed in the organ of Corti sensory and supporting cells, spiral ganglion neurons, lateral wall tissues and cochlear blood vessels. The distribution of adenosine receptors in sensory and neural tissues and the vasculature coincided with other elements of purinergic signalling (P2 receptors, ectonucleotidases), supporting the role of extracellular nucleotides and nucleosides in the regulation of cochlear function [78]. 

Studies on mice with global deletion of A_1_ or A_2A_ receptors demonstrated the distinct roles of these receptors in cochlear physiology and response to injury [77]. Genetic deletion of the A_1_R resulted in early-onset high-frequency hearing loss at ambient sound levels; this hearing loss was aggravated by noise exposure [77]. In contrast, the A_2A_R deletion did not affect auditory thresholds but improved the survival of sensorineural tissues in the cochlea after exposure to traumatic noise. The A_2A_R-null mice demonstrated better preservation of OHC and afferent synapses and minimal loss of SGN after noise exposure. This study suggests that the loss of A_1_R results in increased susceptibility to cochlear neural injury and hearing loss, whilst the absence of A_2A_R increases cochlear resistance to acoustic trauma [77].

Extracellular adenosine concentrations in the mammalian cochlea are regulated by selective adenosine uptake with adenosine transporters and intracellular enzymes such as adenosine deaminase (Ada) and adenosine kinase (Adk) [18] (Figure 2). Two equilibrative (ENT1 and ENT2) and two concentrative (CNT1 and CNT2) nucleoside transporters are expressed in the rat cochlea [80]. Exogenous adenosine perfused through the cochlear perilymphatic compartment was effectively taken up by cells lining this fluid compartment, and the uptake of adenosine was inhibited by an adenosine uptake blocker nitrobenzylthioinosine. The study demonstrates the bi-directional nucleoside transport in the cochlea, supporting adenosine recycling and regulating adenosine concentrations in cochlear fluid spaces [80].

Intracellularly, adenosine is hydrolysed by Ada to inosine, whilst Adk phosphorylates adenosine to adenosine 5′-monophosphate (AMP) [18]. It was reported that Ada-deficient patients have bilateral SNHL and severe immune deficiency, which were faithfully reproduced in *Ada*-null mice [81]. Ada deficiency in those mice was associated with hearing deficits and damage to cochlear hair cells, but early initiation of enzyme replacement therapy improved hearing and immune abnormalities [82]. Interestingly, elevated cochlear adenosine levels in untreated mice were associated with enhanced expression of *Adora2b* gene encoding A_2B_R. Treatment with an A_2B_R antagonist significantly improved hearing loss in *Ada*-null mice, nerve fiber and myelin density, suggesting that the activation of A_2B_R aggravates sensorineural hearing loss (SNHL) [81]. 

## 8. Adenosine A_1_ Receptors and Sensorineural Hearing Loss

Hearing loss is a global health issue. The World Health Organization estimates that by 2050, over 700 million people, or one in every ten people, will experience disabling hearing loss [83]. Noise-induced hearing loss (NIHL) has become a leading occupational health risk in developed countries and may result from unsafe recreational, social, and residential noise exposures. However, pharmacological treatments for NIHL are still lacking.

Adenosine is a constitutive cell metabolite with an established role in tissue protection and regeneration. The adenosine A_1_ receptors are the primary mediators of cytoprotection in the cochlea [18]. The activation of A_1_R protects from hearing loss by inhibiting oxidative stress, inflammation and apoptotic pathways in the cochlea [76]. The overproduction of reactive oxygen species (ROS) induces expression of the A_1_R via activation of the nuclear factor kappa B (NF-kB) [84]. Mice with genetic deletion of the NF-kB p50 subunit demonstrate altered expression of A_1_R and A_2A_R and distinctive behavioral phenotypes, suggesting a critical role of NF-kB in expression levels of adenosine receptors [85]. However, exogenously administered adenosine receptor agonists are required to boost the protective capacity of these receptors under oxidative stress conditions.

Previous studies have shown that A_1_R agonists can prophylactically reduce noise-induced cochlear injury. For example, intratympanic administration of A_1_R agonist R-phenylisopropyladenosine (R-PIA) before acoustic overexposure significantly improved auditory thresholds and hair cell survival in the chinchilla cochlea [86]. 

However, the post-exposure treatment of NIHL is more appealing from a clinical perspective. Wong et al. demonstrated, for the first time, that A_1_R agonists (adenosine, 2-chloro-N-cyclopentyladenosine or CCPA), applied to the round window membrane of the cochlea 6 hours after noise exposure, effectively reduced auditory brainstem threshold shifts in rats by reducing oxidative stress and noise-induced hair cell loss [87]. In contrast, the selective A_2A_R agonist CGS-21680 and A_3_R agonist Cl-IB-MECA did not protect the cochlea from injury and hearing loss [87].

More recently, a selective A_1_ adenosine receptor agonist, adenosine amine congener (ADAC), emerged as a potentially effective treatment for noise-induced cochlear injury and hearing loss [88,89,90]. The post-exposure treatment with ADAC led to a significantly greater recovery of hearing thresholds and improved survival of sensory hair cells in rats compared with non-treated controls [89]. We have also demonstrated the dose-dependent rescue effect of ADAC on noise-induced cochlear injury and established the time window for treatment [88]. ADAC was most effective in the first 24 hours after noise exposure (8–16 kHz, 110 dB sound pressure level for 2 hours), providing up to 21 dB protection averaged across the frequencies (8–28 kHz). The drug was effective at doses 50–200 μg/kg administered as five consecutive daily intraperitoneal injections. Even delayed treatment 48 hours after noise exposure provided clinically significant improvement of auditory brainstem thresholds (>10 dB) at some frequencies [88]. These data show that ADAC mitigates noise-induced hearing loss in a dose- and time-dependent manner and support ADAC development as a potential clinical otological treatment for acute sensorineural hearing loss caused by exposure to traumatic noise. 

The chemotherapeutic agent cisplatin can also cause the upregulation of adenosine receptors in the cochlea, which likely represents a compensatory mechanism to counter the toxic effects of cisplatin-induced ROS overproduction [91]. More recent studies implicate ROS-induced inflammatory and apoptotic processes in the cochlea by activating signal transducer and activator of transcription (STAT1) [79]. A_1_R activation protects against cisplatin ototoxicity by suppressing inflammatory and oxidative stress responses initiated by ROS generation [79]. Intratympanic or parenteral administration of the A_1_R agonists R-PIA, CCPA and ADAC significantly reduced cisplatin-induced threshold shifts and protected against cisplatin-induced hair cell damage [92,93]. These studies suggest that the A_1_R contributes significantly to cochlear protection from ototoxic drugs and mitigates drug-induced hearing loss.

In contrast, inhibition of the A_1_R by a broad spectrum adenosine receptor antagonist caffeine potentiated cisplatin-induced hearing loss in a rat model of cisplatin ototoxicity [94]. A single-dose oral administration of caffeine exacerbated cisplatin-induced hearing loss by increasing synaptopathy and inflammation in the cochlea, whereas multiple doses of caffeine were associated with enhanced damage to OHC. This study suggests that caffeine consumption should be limited in cancer patients treated with cisplatin [94].

Aminoglycoside antibiotics can also cause sensorineural hearing loss [95]. Using an established organotypic tissue culture model of the neonatal mouse cochlea, Lin et al. investigated the effect of P1 (adenosine) and P2 (ATP) receptor activation on the sensory hair cell survival after exposure to the ototoxic aminoglycoside neomycin [96]. Neomycin-induced ototoxicity was aggravated by the addition of slowly hydrolyzable ATP analog ATPγS, whilst the activation of adenosine receptors by ADAC or adenosine conferred partial protection from neomycin ototoxicity. It was inferred that adenosine A_1_ receptors are critical for maintaining cochlear homeostasis and survival following ototoxic injury [96].

## 9. Adenosine Kinase and Age-Related Hearing Loss

Age-related hearing loss (ARHL) is the most common sensory disability, characterized by a decline in hearing sensitivity and speech discrimination, impaired sound localization, and delayed auditory information processing [97]. Currently, there are no treatments that can prevent or delay ARHL. A preclinical study [98] demonstrated that enhanced adenosine receptor signalling in the cochlea could provide partial protection from ARHL in C57BL/6J mice characterized by accelerated hearing loss [99]. That study [98] targeted adenosine kinase (Adk), the key enzyme in adenosine metabolism that regulates intracellular and extracellular adenosine concentrations, using a selective Adk inhibitor ABT-702 administered intraperitoneally twice a week, from three to nine months of age. At nine months, mice treated with ABT-702 showed lower auditory brainstem threshold shifts and improved survival of sensory hair cells compared to control non-treated mice. The study provides the first evidence that ARHL can be partially prevented or delayed by enhancing endogenous adenosine receptor signalling in the cochlea [98].

## 10. Regulators of G Protein Signalling and Hearing Loss

Previous studies have shown that the “post-exposure” treatment with adenosine A_1_R agonists confers partial protection against acoustic trauma and other forms of SNHL [87,88]. Another study attempted to rescue noise-induced cochlear injury by post-exposure treatment targeting A_1_R without using receptor agonists [100]. This was achieved by pharmacological inhibition of the protein that regulates G protein signalling by the A_1_R (Regulator of G protein Signalling 4, RGS4). A molecular complex between RGS4 and neurabin, an intracellular scaffolding protein expressed in neural and cochlear tissues, is the key negative regulator of A_1_R activity [101]. In that study, Wistar rats (6–8 weeks) were exposed to traumatic noise (110 dB SPL, 8–16 kHz for 2 h), and a small molecule RGS4 inhibitor CCG-4986 was delivered by intratympanic injection 24 or 48 h after noise exposure. Intratympanic administration of CCG-4986 48 h after noise exposure attenuated noise-induced auditory threshold shifts by up to 19 dB, whilst the earlier drug administration (24 h) led to even better preservation of auditory thresholds (up to 32 dB). Functional improvement was linked to improved survival of sensorineural tissues and afferent synapses in the cochlea. RGS inhibitors thus represent a novel paradigm for treating noise-induced cochlear injury and possibly other forms of SNHL [100].

Subsequent studies have shown that other members of the RGS family of proteins may be involved in cisplatin-induced ototoxicity. Cisplatin upregulates RGS17 expression in the cochlea, leading to increased oxidative stress, upregulation of inflammatory genes and enhanced apoptosis in the cochlea [102]. The silencing of *RGS17* suppressed cisplatin-induced hearing loss in rats, while overexpression of *RGS17* enhanced cisplatin-induced hearing loss. RGS17 represents a novel mediator of cisplatin ototoxicity and a potential therapeutic target for cisplatin-induced hearing loss [102].

## 11. Adenosine A_2A_ Receptors and Hearing Loss

The role of A_2A_ receptors in cochlear injury development appears to be opposite to the A_1_R. Previous studies in mice with genetic deletion of the adenosine A_2A_ receptor have demonstrated better preservation of cochlear afferent synapses and spiral ganglion neurons after acoustic overexposure compared to control wildtype mice [77]. This has informed our alternative approaches to cochlear neuroprotection based on pharmacological inhibition of the A_2A_R. In a rat organotypic tissue culture model of excitotoxic injury (combined exposure to NMDA and kainic acid), the co-administration of istradefylline (a clinically approved A_2A_R antagonist) reduced deafferentation of the inner hair cells and improved the survival of afferent synapses after excitotoxic injury [103]. This study may have implications for the treatment of cochlear neuropathy and the prevention of hidden hearing loss as its clinical manifestation.

**Figure 4:**
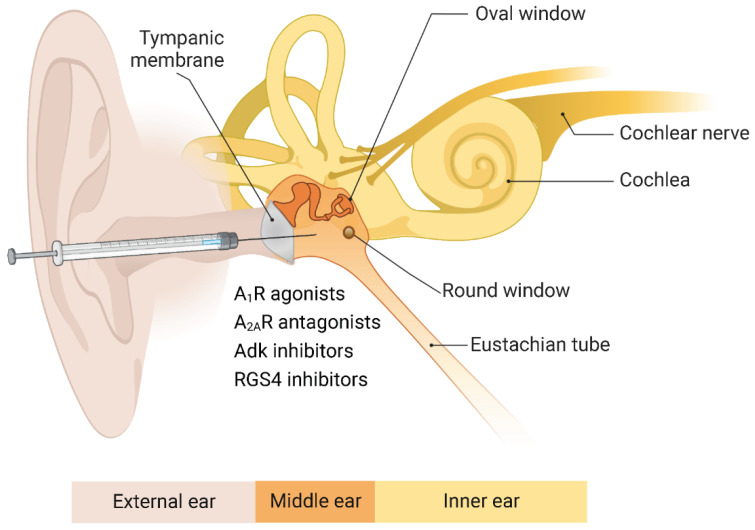
**Cochlear protection by adenosine receptors.** The preclinical studies established four distinct strategies targeting adenosine receptors to rescue the cochlea from injury and prevent sensorineural hearing loss. These pharmacological interventions include the administration of A_1_ receptor agonists, A_2A_ receptor antagonists, adenosine kinase (Adk) inhibitors and RGS4 (Regulator of G protein signalling 4) inhibitors. The most common route for drug delivery to the inner ear is the intratympanic injection onto the round window membrane of the cochlea, which precludes off-target effects associated with systemic administration. Created with BioRender.com.

The A_2A_ receptor targeting may also be relevant for the preventative treatment of age-related hearing loss. Middle-aged C57BL/6J mice, prone to early onset ARHL, were given weekly istradefylline injections (1 mg/kg) from 6 to 12 months of age [104]. Auditory function was assessed using ABR to tone pips (4–32 kHz) at 6, 9, and 12 months of age. Weekly injections of istradefylline attenuated ABR threshold shifts by approximately 20 dB at mid to high frequencies (16–32 kHz) and improved hair cell survival in a turn-dependent manner. This study presents the first evidence for the rescue potential of istradefylline in ARHL [104]. This and other options for the therapeutic targeting of adenosine receptors in the cochlea are shown in Figure 4.

## 12. Conclusions

Purinergic signalling is an intricate system of extracellular receptors, enzymes and transporters that regulates multiple physiological and pathophysiological processes in the mammalian inner ear. P2 receptors have an essential role in cochlear development, regulation of electrochemical homeostasis, auditory neurotransmission, and adaptation to elevated sound levels. Surface-located ectonucleotidases (NTPDases and CD73) hydrolyse extracellular ATP to adenosine and thus convert the P2 receptor environment into the P1 (adenosine) receptor environment. The two main types of adenosine receptors, A_1_ and A_2A_, have differential roles in cochlear injury response, highlighting their clinical significance as prospective therapeutic targets. Pharmacological manipulation of P2 receptor signalling is also a developing strategy for the therapeutic management of hearing loss.
